# Psychometric properties of a Power Mobility Caregiver Assistive Technology Outcome Measure

**DOI:** 10.1371/journal.pone.0178554

**Published:** 2017-06-06

**Authors:** W. Ben Mortenson, Louise Demers, Paula W. Rushton, Claudine Auger, François Routhier, William C. Miller

**Affiliations:** 1 Department of Occupational Science and Occupational Therapy, Faculty of Medicine, University of British Columbia, Vancouver, British Columbia, Canada; 2 International Collaboration on Repair Discoveries, Research Lab, Vancouver, British Columbia, Canada; 3 GF Strong, Rehabilitation Research Program, Vancouver, British Columbia, Canada; 4 École de réadaptation, Université de Montréal, Montreal, Quebec, Canada; 5 Centre de recherche de l'Institut universitaire de gériatrie de Montréal, Montreal, Canada; 6 CHU Sainte-Justine Research Centre, Montreal, Quebec, Canada; 7 Center for Interdisciplinary Research in Rehabilitation, Montreal, Quebec, Canada; 8 Département de réadaptation, Université Laval, Quebec City, Quebec, Canada; 9 Center for interdisciplinary research in rehabilitation and social integration, CIUSSS de la Capitale-Nationale, Institut de réadaptation en déficience physique de Québec, Quebec City, Quebec, Canada; 10 Centre intégré de santé et de services sociaux de la Capitale-Nationale, Quebec, Canada; 11 Institut de réadaptation en déficience physique de Québec, Quebec City, Quebec, Canada; Universidade Federal do Rio de Janeiro, BRAZIL

## Abstract

Caregiver burnout is a serious concern among informal caregivers, especially for those who provide care to individuals with more severe limitations such as power mobility users. The Power Wheelchair Caregiver Assistive Technology Outcome Measure tool measures device specific and overall burden experienced by informal caregivers of power mobility users. A one-month, test-retest study was conducted to examine the reliability, internal consistency, and construct validity of the Power Wheelchair Caregiver Assistive Technology Outcome Measure. Two construct validity measures were administered: the Hospital Anxiety and Depression Scale and the Late Life Disability Index. The test-retest-reliabilities of part 1 (power wheelchair specific burden) and part 2 (general caregiving burden) were 0.769 and 0.843 respectively. Scores on part 1 were moderately and positively correlated with part 2 and with frequency of participation. Scores on part 2 were moderately and negatively correlated with anxiety, depression, and positively with perceived limitation of participation. The strength and direction of these correlations provide support for the construct validity of the measure and suggest part 1 and part 2 provide complementary information. Further testing is needed to assess the clinical utility and responsiveness of the measure.

## Introduction

Power wheelchairs are a common form of assistive technology. In 2010, it was estimated that about 3.6 million of those over the age of 15 in the United States used a wheelchair for mobility assistance [1]. It is estimated that approximately 15% of these use power wheelchairs for mobility [2]. Power wheelchairs are typically used by those who have difficulty mobilizing independently using a manual wheelchair. Therefore, power mobility users tend to have lower levels of functional independence than manual wheelchair users and likely need additional assistance from caregivers.

Approximately 80% of the assistance that is required by people with disabilities is provided by informal caregivers (e.g., friends, family, neighbours) [3]. Informal caregivers provide unpaid assistance for basic and instrumental activities of daily living to individuals who are ill or have disabilities. The majority (65%) of informal caregivers are women and more than 80% provide assistance to a relative or close friend over the age of 50 [4]. With over 50 million informal caregivers in the United States, the economic contributions of this care were estimated at $450 billion USD in 2009 [4]. Informal caregivers often experience high stress, feelings of depression and anxiety, decreased social participation and isolation [5–7]. Caregiver burnout, described as physical, mental or emotional exhaustion, is a serious concern with informal caregivers.

Provision of assistive technologies has been identified as a potential mechanism to reduce the need for assistance and potentially reduce caregiver burden [8, 9]. For example, studies have reported that provision of power mobility resulted in decreased a) need for assistance with outdoor mobility, b) numbers of transfers for which assistance was required, c) perceived need for mobility supervision [9–11]. However, the increased weight of powered wheelchairs makes transportation more challenging and the technical complexity of power wheelchairs may necessitate additional training for caregivers of power mobility users [12]. Several negative outcomes of power wheelchair use on caregivers have also been noted. These include potential caregiver injury, caregiver anxiety about user injury, and accessibility challenges limiting the locations available to visit (e.g., manual wheelchairs can negotiate higher curbs than most power wheelchairs) [8–11, 13].

In order to capture the caregiver burden related to the provision of power wheelchair assistance, the Power Wheelchair Caregiver Assistive Technology Outcome Measure (CATOM-PW) was developed. This measure was adapted from the generic version of the Caregiver Assistive Technology Outcome Measure, which was created for use in an experimental study [14]. The original measure included two parts. In part 1, after reviewing all of the care they provided, respondents were asked to identify their most burdensome caregiving activity. This activity was then targeted for intervention. In part 1, respondents answered 14 questions about specific areas of burden associated with activity (e.g., need to provide assistance, risk to personal safety). In the second part, caregivers responded to four questions related to their overall burden (i.e., the impact on their personal activities (e.g. work; leisure) and relationships). For the original measure, the 6-week, test-retest intraclass correlation coefficients were 0.88 for part 1 and 0.86 for part 2 [14]. The CATOM scores were correlated as hypothesized with measures of care recipients’ independence performing activities. [14].

The modified CATOM-PW similarly consists of 2 parts with 18 items in total. In part 1 (14 items), caregivers rate the burden that they experience with different aspects of the provision of wheelchair assistance. In part 2, caregivers answer four questions related to their overall perceived burden. The CATOM-PW differs from the original, in that instead of asking caregivers to complete part 1 as they reflect on their most problematic self-identified caregiving activity, it asks specifically about burden related to power wheelchair related tasks, which include transfers, maintenance, steering the power wheelchair inside, transporting the power wheelchair, driving the power wheelchair outside and operating special wheelchair features. Furthermore, in part 2, caregivers are asked to identify the types of assistance they provided (i.e., eating, washing, dressing, grooming, toileting, mobility, transfers (non-wheelchair), walking inside, negotiating stairs, getting around outside without wheelchair, housekeeping, meal preparation, shopping, laundry, telephone, transportation, medication use, budgeting, leisure, and work activities), which had previously been noted in part 1 of the original measure.

Given that no psychometric testing had been conducted with the modified measure, a study was undertaken with three aims related to the measure: 1) to assess the test-retest reliability, 2) to determine the internal consistency and 3) to evaluate the construct validity. CATOM-PW. In terms of its construct validity, we anticipated part 1 of the CATOM-PW would be moderately correlated with measures of depression and anxiety and frequency of participation and lack of perceived limitations to participation, given previous research which has found that caregiver burden is associated with increased anxiety and depression [15] and decreased activity participation [16]. Given the more global assessment of perceived burden of part 2 of the CATOM-PW, we hypothesized that it would be correlated in the same direction, but with greater strength with the same measures and would be moderately correlated with part 1 of the CATOM-PW, as was found with the original measure [14]. This is based on the assumption that power wheelchair-related caregiving activities would represent a smaller subset of all caregiving activities.

## Methods

This study involved a test-retest design consisting of baseline measurement and follow-up one month later. These data were collected as part of a larger longitudinal multi-site study. The six Canadian sites included Halifax, Nova Scotia; London, Ontario; Montréal, Québec; Québec City, Québec and Toronto, Ontario; Vancouver, British Columbia. The study received ethics board approval in each of the jurisdictions in which the research was conducted. Approval for the primary site was obtained from the University of British Columbia, Office of Research Sciences, Behavioral Research Ethics Board (UBC BREB NUMBER: H10-00214).

### Sample

To be included in the study, informal caregivers needed to 1) provide care to a power wheelchair user who was over the age of 50, 2) be able to understand either French or English, and 3) be able to provide independent consent (e.g., competent and over the age of 18 or 19 depending on the jurisdiction).

### Study recruitment

Recruitment of participants for this study was conducted between May 2010 and December 2012. Informal caregivers were recruited from rehabilitation centers, wheelchair seating programs (i.e. health-care services which specialize in the measurement and provision of wheelchairs and wheelchair seating), and customer lists from wheelchair equipment vendors. Other strategies included the use of third party recruiters, social media posts and advertisements in newspapers. Interested participants provided the vendor or program facilitator with their contact information and verbal consent to be contacted by the research coordinator at the local site. The research coordinator then provided study details, answered questions and confirmed participant eligibility for the study. All measures were available in French and English. Once eligibility was confirmed, appointments were scheduled for data collection.

### Descriptive data

Demographics, training background and care provision information were collected for each participant via self-report and included: year of birth, age, gender, primary language, marital status, highest education level, annual household income, relationship to power wheelchair user, living arrangement, frequency of assistance in maneuvering power wheelchair, employment status, receipt of formal training for care provision, receipt of formal wheelchair skills training, and hours and frequency of care provided.

### Measures

The main measure of interest was the CATOM-PW. In the first section of the first part 1CATOM-PW, caregivers identify all of the power wheelchair related tasks for which they provide assistance via an open ended question and six closed questions inquiring specifically about 1) transfers to and from the wheelchair, 2) wheelchair maintenance, 3) propelling the power wheelchair inside, 4) transporting the power wheelchair (e.g., up/ down stairs/ into/ out of vehicle), 5) driving the power wheelchair outside and 6) operating special wheelchair features (e.g., tilt-in-space, elevating leg rest, recline). In the second section of part 1, caregivers rate how frequently they experience burden in 14 different aspects of these power wheelchair related caregiver activities (e.g., physical demands, worry, time demands) using a five point Likert scale (5 = never and 1 = nearly always). In the first section of part 2, caregivers identify the other caregiving activities they perform via an open-ended question and 16 closed questions (based on Functional Autonomy Measure items [17]). In the second section of part 2, caregivers rate frequency of burden they experience with all their caregiving activities (e.g., overall burden) on 4 items (e.g., leisure, work, social relationships) using the same response scale as the second section of part 1. This measure was administered at baseline and at one-month follow-up.

The Hospital Anxiety and Depression Scale (HADS) is a self-report scale that assesses anxiety and depression through 14-items measured on a 4-point scale (0–3) (7-items for each construct) [18]. Higher scores are indicative of higher symptom frequency (e.g. most of the time or definitely). This measure has been demonstrated to have good construct validity (e.g., total score was negatively correlated to life satisfaction) and internal consistency in people living with spinal cord injury [19].

The Late Life Disability Index (LLDI) was used to measure the frequency and perceived limitation of participation among caregivers in the study. Frequency is measured on a five-point scale (i.e., very often, often, once in a while, almost, never and never) and perceived limitations is measured on a similar five-point scale (i.e., not at all, a little, somewhat, a lot, completely). It records data about 16 life tasks (e.g., keeping in touch with others, preparing meals, active recreation, and taking care of errands). Summative scores are calculated for each domain and higher scores indicate increased frequency and lower levels of perceived limitations.

### Procedure

Written consent was obtained by the rater from each participant during the initial visit (research was approved by The University of British Columbia, Office of Research Sciences, Behavioral Research Ethics Board. UBC BREB NUMBER: H10-00214). During the first visit at the research center, baseline data were collected on all three measures described above. Four-week re-test measures were conducted by the rater.

### Analysis

All data collected were screened for accuracy and completeness by research coordinators at each site and by the overall project coordinator. All analyses were conducted in Statistical Package for Social Sciences (SPSS) version 22. Descriptive statistics were used to summarize measure scores and demographic variables. Mean and standard deviations were computed for continuous variables, e.g. scores for each measure, while proportions were calculated for categorical variables, such as gender.

For the first aim, intra-class correlation coefficients (ICCs) (2, 1) were used to evaluate the reliability of the CATOM-PW between the baseline test and one month follow-up retest. An ICC between ≤0.75 and ≥0.40 is considered moderate, while an ICC >0.75 is excellent [20]. For the second aim, Cronbach’s alpha was used to assess internal consistency of the CATOM-PW. A value >0.8 is considered strong and <0.8 >0.7 is considered moderate [20]. For the third aim, the validity of the CATOM-PW was assessed using Pearson’s correlation coefficients. Data from the baseline tests were used for validity purposes. A correlation was considered moderate if it was between 0.3 and 0.6 [21].

## Results

Table 1 provides demographic information about the sample and their scores on the CATOM-PW at both time points and construct validity measures at baseline. The sample was predominantly female, and most were unemployed. More than half of the sample provided informal care to their spouse. Only one caregiver reported receiving formal wheelchair skills training (self-identified). | (Table 1) The mean time between time points was 35 days (SD = 11.1). Caregivers assisted with an average of 11 (SD = 3.3) power mobility related tasks. One subject returned the questionnaires by mail, three subjects completed the questionnaires via telephone, and four subjects completed the questionnaires via mail and telephone.

**Table 1:** Descriptive statistics for caregivers’ demographic characteristics and measures.

Variables (Range for Standardized Measures)	Sample Mean ± SD or N [%]
Age (years)	63.69 ± 10.15
Female	23 [65.7]
Language	
English	25 [71.4]
French	6 [17.2]
Other	4 [11.6]
Marital Status	
Single/Never Married	4 [11.4]
Married	26 [74.3]
Widowed	3 [8.6]
Education	
Primary and Elementary	1 [2.9]
High School	11 [31.4]
College or Trade School	11 [31.4]
University/Postgraduate Studies	12 [34.3]
Annual Income	
< 14 999	2 [5.7]
15 000–29 999	10 [28.6]
30 000–44 999	5 [14.3]
60 000–74 999	5 [14.3]
> 75 000	6 [17.1]
Relationship with wheelchair user	
Spouse	27 [77.2]
Family member	2 [5.7]
Friend	6 [17.1]
Live in same residence	
Yes	27 [77.1]
No	8 [22.9]
Frequency of assistance (physically, verbally) with maneuvering powered wheelchairs	
Not yet occurred	3 [8.6]
Daily	21 [60]
Weekly	10 [28.6]
Monthly	1 [2.9]
Employment Status	
Employed	8 [22.9]
Unemployed	27 [77.1]
Retired	13 [37.1]
Other	8 [22.9]
Formal Training for care	
Yes	1 [2.9]
No	34 [97.1]
Formal wheelchair skills training	
Yes	1 [2.9]
No	34 [97.1]
LLDI Frequency (0–100)	57.0 ± 6.48
LLDI Limitation (0–100)	59.2 ± 12.0
HADS Anxiety (0–21)	6.9 ± 4.31
HADS Depression (0–21)	4.7 ± 3.20
Baseline CATOM-PW part 1 (14–70)	53.8 ± 9.48
Baseline CATOM-PW part 2 (4–20)	13.4 ± 4.49
Follow up CATOM-PW part 1 (14–70)	54.0 ± 8.19
Follow up CATOM-PW part 2 (4–20)	14.4 ± 4.41

### Reliability and internal consistency (Aims 1 and 2)

Table 2 presents the reliability estimates and the internal consistency of parts 1 and 2 of the CATOM-PW. The ICCs and Cronbach’s alpha were >0.75 for both parts of the CATOM-PW. (Table 2)

**Table 2:** Test-retest reliability and internal consistency for part 1 and part 2 CATOM-PW scores.

Dimension	N	Intraclass Correlation	95% confidence interval		Cronbach’s α
			Lower Bound	Upper Bound	
CATOM-PW part 1	31	.769	.573	.881	.756
CATOM-PW part 2	31	.843	.700	.921	.788

### Validity (Aim 3)

The correlations between the CATOM-PW and other measures in the study are provided in Table 3. Scores on part 2 were correlated with other measures as hypothesized (i.e., participants who perceived less overall burden, reported fewer symptoms of anxiety and depression, fewer limitations and greater frequency of participation). Scores on part 1 were correlated as anticipated with part 2 and were moderately correlated with frequency of participation (i.e., the less power wheelchair related burden participants perceived the greater their frequency of participation). Scores on part 1 were only weakly correlated with other measures, but these correlations were in the same direction as correlations with part 2. (Table 3)

**Table 3:** Correlations among part 1 and part 2 CATOM-PW scores and other variables.

Variable	CATOM-PW part 1	CATOM-PW part 2	LLDI Frequency	LLDI Limitation	HADS Anxiety	HADS Depression
CATOM-PW part 1						
Pearson	1	.616	.313	.107	-.120	-.102
Significance		<.001	.081	.560	.500	.567
N	34	34	32	32	34	34
CATOM-PW part 2						
Pearson	.616	1	.329	.405	-.352	-.356
Significance	<.001		.066	.021	.041	.039
N	34	34	32	32	34	34

## Discussion

This is the first paper to explore the measurement properties of the CATOM-PW. This work builds on research on original version of the CATOM [14].

As was found with the original version of the measure, the CATOM-PW demonstrates excellent test-retest reliability. However, the ICCs for part 1 are lower than those reported for the original version of the CATOM (e.g., 0.769 in the current study, compared to 0.860 with the original CATOM). This may be due to the identification and evaluation of only one caregiving activity (e.g., toilet transfers, or bathing) with the original CATOM, whereas the CATOM-PW requires respondents to consider a variety of power mobility related caregiving activities. It may also reflect real changes in caregiver burden, which may have been reduced if a shorter period (e.g., two weeks) was used between administrations.

The CATOM-PW demonstrates moderate internal consistency, which is considered acceptable [22]. This likely reflects the variety of items included, which may reduce between item correlations. A larger sample would enable an exploration of how the items load on one another to identify if any sub-scales scores should be developed) and further evaluate the relationship between part 1 and part 2 items.

As hypothesized, scores on part 1 and part 2 of the CATOM-PW were moderately correlated [14]. Likewise, although not significant likely, due to the small sample size, scores on part 1 of the CATOM-PW were moderately correlated with LLDI frequency of participation. This makes intuitive sense given that decreased power mobility related caregiver burden may enable caregivers to participate in other activities more frequently. The measure was not significantly correlated with other measures, which may be due to the targeted nature of power mobility specific assistance, although it should be noted that the correlations were in the same directions as those found for part 2 of the measure.

Part 2 of the CATOM-PW varied as anticipated with other measures. Not surprisingly, there was a significant negative correlation of moderate strength with both subscales of HADS, suggesting there may be increased feelings of anxiety and depression with more caregiver burden. The relationship between caregiver burden and depression and anxiety is not unexpected given previous studies that have similarly relationships between these variables [7, 23]. As hypothesized, part 2 was moderately correlated with LLDI limitation, as increased caregiver involvement and time would limit participation in other activities unrelated to providing care. This is in keeping with previous research, which found a negative correlation between caregiving burden and participation in meaningful activities [24].

Additional research would be beneficial to further validate the CATOM-PW. This could include studies with different samples (e.g., caregivers who work; non-spousal caregivers, male caregivers). A larger sample size would enable factor analysis to be undertaken to examine the dimensionality of the tool. Intervention studies could be used to evaluate the responsiveness of the measure.

### Limitations

There are three main limitations to the study. First, a social desirability bias may have affected the self-report measures included in the study. For example, this may have altered the construct validity correlations, if caregivers downplayed the negative effects of care provision. Second, the small sample size increases the confidence intervals. Finally, given that a sample of convenience was used, the results may not be generalizable to other caregivers of power mobility users.

## Conclusion

A research study was undertaken to evaluate the psychometric properties of the CATOM-PW, a measure that was designed to identify caregiver burden associated with the provision of power mobility and assist in the evaluation of power mobility related interventions. The CATOM-PW demonstrates excellent one month, test-retest reliability, and internal consistency. The first part varies as anticipated with the latter half of the measure and with frequency of participation and the second part of the measure varies as anticipated with other measures. Overall, the psychometric properties of the study appear promising, but further research would be beneficial to further validate the measure and to explore how the tool can be used clinically.
